# Obstetrics, Gynecology and Abdominal Surgery

**Published:** 1901-12

**Authors:** William Warren Potter

**Affiliations:** Buffalo, N.Y.


### PROGRESS IN MEDICAL SCIENCE.

### Obstetrics, Gynecology and Abdominal Surgery.

Conducted by WILLIAM WARREN POTTER, M.D., Buffalo, N.Y.

### DIAGNOSIS, ETIOLOGY, PROPHYLAXIS AND TREATMENT OF CYSTITIS, PYELITIS AND PYELONEPHRITIS IN WOMEN.

T. R. Brown (*New York Med. Jour.*, Vol. 74, No. 9,) thus summarises an excellent article:

1. The great majority of cases of pyelitis, pyleonephritis and cystitis are due to infection with various microörganism (of which the colon bacillus is the commonest) which may reach the kidney or bladder either exogenously or endogenously.

2. That in the majority of cases the condition either can be prevented or can be cured if the conditions underlying its development are recognised and understood, and the correct mea sures inaugurated.

3. There are various conditions, such as urinary hyperacidity, which may simulate almost exactly true vesical infections, and yet in which misinterpretation and improper treatment may lead to the development of a true cystitis and its deplorable consequences.

4. In no condition is prophylaxis more essential than in that of the infections of the urinary tract, while to be able to prevent such conditions we must have constantly before us the danger of the development of infection in all cases associated with conditions which tend to lower the general resistance of the patient, and also those which render the bladder susceptible to infection, especially by the trauma of an operation or catheterism.

5. While an absolute diagnosis of renal infection can be made only by ureteral catheterism, in the majority of cases a very probable diagnosis may be arrived at by a consideration of the relation between the grade of albuminuria and pyuria and by careful cystoscopic examination of the bladder, especially that portion about the ureteral orifices, and the character of the urine flowing therefrom.

6. Contrary to the opinion expressed in the majority of text-books, a great majority of the infections both of the bladder and of the kidney are associated with acidity of the urine—that is, are due to microörganisms which do not split up the urea.

7. Probably in the majority if not in all the cases of renal affection due to a urea-decomposing microörganism, after the condition has lasted for a certain length of time, a stone is formed by the decomposition of the precipitated salts about the bacteria as a nucleus.

8. And, finally, to be able to thoroughy understand the cases of cystitis, pyelitis and pyelonephritis brought to our notice, to make the proper diagnosis, to inaugurate and carry out a rational line of treatment, to be conversant with the proper means of prophylaxis, and to give a correct prognosis, a careful chemic, microscopic and bacteriologic study of the urine is absolutely essential.

### THE COMPLICATIONS AND DEGENERATIONS OF FIBROID TUMORS OF THE UTERUS AS BEARING UPON THE TREATMENT OF THOSE GROWTHS.

C. P. Noble (*Am. Jour. of Obstet.*, Vol. 44, No. 3,) in concluding a paper with the above title, says:

It is gratifying to contrast the results which can be secured through the resources of modern gynecology with those which would follow an expectant plan of treatment. The mortality of hysterectomy and myomectomy is variously estimated at from 2 to 10 per cent. In a series of 345 cases published by myself in 1897, the mortality of hysterectomy by supravaginal amputation in the hands of five American gynecologists was 4.9 per cent.; in a series of 100 total hysterectomies the mortality was 10 per cent. In a collection by Olshausen of 806 cases of supravaginal amputation, the mortality was 5.6 per cent., contrasted with a mortality of 9.6 per cent. in a collection of 520 cases of total extirpation. According to Bishop, Mr. Christopher Martin reports 35 cases of total extirpation, with a mortality of 2.8 per cent.; Doyen 60 cases, with a mortality of 2.6 per cent.; A. Martin 81 cases, with a mortality of 7.4 per cent. The advocates of vaginal hysterectomy for fibroid tumors report equally as good, if not better results.

The results of myomectomy indicate that enucleation is a more dangerous operation than hysterectomy, although in the hands of trained men the results are excellent. Kelly reports 97 myomectomies, with 4 deaths. This is to be contrasted with 307 hystero-myomectomies, with 15 deaths, or a mortality of 4.8 per cent. MacMonagle reports 65 cases of myomectomy, with no death.—*Memphis Med. Monthly.*

---

### DIFFICULT POINTS IN GYNECOLOGIC DIAGNOSIS.

After alluding to the difficulties of gynecologic examinations in many cases, Krusen (*Phil. Med. Jour.*) discusses the subject of the diagnosis of uterine carcinoma, and to aid, suggests the following points which will be found useful though they may not be applicable to every case:

1. The usual friability and vascularity of the tissue, which, if not detected by the finger, may easily be made apparent by hooking a tenaculum into the suspected area. The tenaculum will immediately tear out and cause abundant bleeding from the carcinomatous tissue.

2. A close adhesion of the mucous membrane of the portio to the parenchyma.

3. The difficulty in cervical dilatation as evidenced by the introduction of a tent. In a cancerous process there is, as a rule, a continuance of the hardness after dilatation.

4. Bleeding is easily provoked by an examination or by any unusual exertion or manipulation.

5. The characteristic induration of the cervix is almost imperceptible at first, but increases as the disease progresses.

6. Puncture of any suspected nodules or follicles will differentiate carcinoma from cystic follicles or distended glands.

7. Ulcerated or eroded areas which are not speedily amenable to treatment should be regarded with suspicion.

8. Any enlargement of the uterus occurring after the menopause, is usually due to malignant disease. (Fisher.)

9. An early diagnosis can only be made with absolute certainty by microscopic examination of either an excised wedge from the suspected cervix, or, in cancer of the body, of portions of the endometrium removed by curetage. The value of the examination will depend upon the experience and competency of the pathologist.

Epithelioma is less frequent, but it is liable to be confused with: (1) simple vegetations; (2) lupus of the vulva; (3) syphilitic affections; but the first, the simple vegetations, secrete a virulent fluid unlike the ichor of cancer, they readily yield to an energetic caustic which prevents their reproduction, and they have no hardened base, being remarkable for their softness. In the case of lupus, the vulva is red and presents scattered fungous ulcerations which are without any indurated base; while the syphilitic chancre is more limited and has a little circle of characteristic indurations. Other points of difficult diagnosis referred to are the ectopic pregnancy, the differentiation of appendicitis from pyosalpinx or ovarian disease; ovarian cysts from ascites, and the perplexing problems of pregnancy complicated with typhoid.

---

### THE VALUE OF CONSERVATIVE TREATMENT OF THE UTERINE APPENDAGES.

A. Goldspohn (*Jour. Amer. Med. Assn.*, August 24, 1901,) says that in view of the extreme value of the conservative treatment of the uterine appendages, and that there are a few who still doubt the success of these methods, he has undertaken a study of the cases upon which he has performed such operations during the past three years. Of 128, it was possible to ascertain the actual condition in 97. Of this number 80 were examined by himself or other physicians, and in 17 cases an accurate and complete written statement by the patient was obtained. Nine were treated by anterior median vaginal celiotomy, 32 by median ventral celiotomy, and 56 by bilateral inguinal celiotomy through the dilated internal inguinal rings in conjunction with an Alexander operation.

In dealing with single or isolated cysts of the ovary the projecting portion is cut away and the lining membrane is peeled or scraped from the remainder, the wound being closed with continuous sutures of fine catgut. Hard and contracted cicatricial parts are occasionally exsected, forming a V-shaped wound, which is closed in the same manner. Multiple cysts near each other, when more deeply situated, are opened by a straight or curved incision on the free border of the ovary and extending usually not more than half-way through it. By this incision all follicles of one centimeter or more in diameter are opened, and the lining is peeled or scraped out, The wound in the ovary is then closed by two sets of sutures, one going to the bottom of the wound and the other coaptating the edges. He uses for this fine catgut, hardened in formalin and sterilised by boiling, but never chromicised. The needles are fine, round, noncutting, and have a full curve.

A tube that contains pus is always sacrificed. In cases where the ovary can be liberated and saved with or without resection, and its tube is closed and but little distended, the latter is walled off with gauze and opened and its contents removed. By suturing a permanent opening is made into the tube, providing its contents are not purulent or tubercular. The value of these conservative methods is widely shown in the results; approximately 85 per cent. were free from pain and depressing nervous and circulatory disorders and enjoying good general health. Pregnancy occurred in 10 per cent. of the cases.—*Medicine.*

